# Race and gender variation in response to evoked inflammation

**DOI:** 10.1186/1479-5876-11-63

**Published:** 2013-03-12

**Authors:** Jane F Ferguson, Parth N Patel, Rhia Y Shah, Claire K Mulvey, Ram Gadi, Prabhjot S Nijjar, Haris M Usman, Nehal N Mehta, Rachana Shah, Stephen R Master, Kathleen J Propert, Muredach P Reilly

**Affiliations:** 1 Cardiovascular Institute, Perelman School of Medicine, at the University of Pennsylvania, 11-136 Smilow Center for Translational Research, Building 421, 3400 Civic Center Boulevard, Philadelphia, PA 19104, USA; 2 Division of Cardiology, University of Minnesota Medical School, Minneapolis, USA; 3 National Heart, Lung and Blood Institute, Bethesda, MD, USA; 4 Division of Pediatric Endocrinology, Children’s Hospital, Philadelphia, PA, USA; 5 Department of Pathology and Laboratory Medicine, Perelman School of Medicine, at the University of Pennsylvania, Philadelphia, PA, USA; 6 Department of Biostatistics and Epidemiology, Perelman School of Medicine, at the University of Pennsylvania, Philadelphia, PA, USA

## Abstract

**Background:**

Race- and gender-variation in innate immunity may contribute to demographic differences in inflammatory and cardiometabolic disease; yet their influence on dynamic responses during inflammatory stress is poorly understood. Our objective was to examine race and gender influence on the response to experimental endotoxemia.

**Methods:**

The Genetics of Evoked Responses to Niacin and Endotoxemia (GENE) study was designed to investigate regulation of inflammatory and metabolic responses during low-grade endotoxemia (LPS 1 ng/kg intravenously) in healthy individuals (median age 24, IQR=7) of European (EA; n=193, 47% female) and African ancestry (AA; n=101, 59% female).

**Results:**

Baseline clinical, metabolic, and inflammatory biomarkers by race and gender were consistent with epidemiological literature; pre-LPS cytokines (e.g. median (IQR) IL-6, 2.7 (2) vs.2.1 (2) pg/ml, P=0.001) were higher in AA than EA. In contrast, acute cytokine responses during endotoxemia were lower in AA than EA (e.g. median (IQR) peak IL-1RA, 30 (38) vs.43 (45) ng/ml P=0.002) as was the induction of hepatic acute-phase proteins (e.g. median (IQR) peak CRP 12.9 (9) vs.17.4 (12) mg/L P=0.005). Further, baseline levels of cytokines were only weakly correlated with peak inflammatory responses (all r_s_ <0.2) both in AA and in EA. There were less pronounced and less consistent differences in the response by gender, with males having a higher AUC for CRP response compared to females (median (IQR) AUC: 185 (112) vs. 155 (118), P=0.02).

**Conclusions:**

We observed lower levels of evoked inflammation in response to endotoxin in AA compared with EA, despite similar or higher baseline levels of inflammatory markers in AA. Our data also suggest that levels of inflammatory biomarkers measured in epidemiological settings might not predict the degree of acute stress-response or risk of diseases characterized by activation of innate immunity.

**Trial registration:**

FDA clinicaltrials.gov registration number NCT00953667

## Introduction

Sustained activation of innate immunity in genetically-prone individuals is a hallmark of sepsis and several chronic inflammatory disorders. In type 2 diabetes mellitus (T2DM) and atherosclerotic cardiovascular disease (CVD), chronic low-grade inflammation also may precede the onset and drive progression of disease [1,2]. However, understanding the interplay of genetic background, demographic characteristics and environmental factors in the regulation of pro- and anti-inflammatory responses to activation of innate immunity remains challenging.

The incidence rates of sepsis and cardiometabolic disease differ by race and gender, with higher incidence in African Americans and males [3-5]. Such differences may be, in part, determined by genomic and epigenomic characteristics that regulate metabolic and inflammatory responses to environmental stresses [6]. For example, differences in the individual response to inflammatory stressors may have important downstream effects on initiation and progression of acute and chronic inflammatory diseases. Epidemiological data have revealed race and gender differences in resting levels of inflammatory biomarkers [7,8] and some of these biomarkers are predictors of the subsequent development of cardiometabolic disease [9,10]. Little is known, however, of demographic influences on the response of such biomarkers to inflammatory stresses.

We and others utilize a low-dose endotoxemia model for the study of inflammatory, metabolic and cardiovascular perturbations in humans [11-16]. The endotoxin lipopolysaccharide (LPS) is the exogenous ligand for toll-like receptor-4 (TLR4), activating innate immunity and recruiting adaptive responses. TLR4 signaling is a key link between exogenous and endogenous innate immune antigens and downstream metabolic complications of chronic inflammation in obesity, insulin resistance and atherosclerosis [17]. In rodent models, absence of TLR4 confers protection against diet-induced obesity, insulin resistance [18] and atherosclerosis [19]. In human sepsis and experimental endotoxemia, lipoprotein and metabolic abnormalities emerge that parallel those observed chronically in cardiometabolic diseases [11,12,14,20,21]. Genetic variation in TLR4 that modulates inflammatory response has been associated with reduced risk of atherogenesis [22] supporting the relevance of LPS and TLR4 signaling to chronic cardiometabolic disease. Thus, endotoxemia serves as a probe of innate immune signaling in multiple human pathophysiologies including infection, sepsis and complex cardiometabolic pathophysiologies.

The Genetics of Evoked Responses to Niacin and Endotoxemia (GENE) study was designed to investigate the genomic basis of inflammatory and metabolic responses to endotoxemia and pharmacological doses of nicotinic acid in healthy individuals of European and African ancestry. In this report, we address the hypothesis that the systemic inflammatory responses to LPS would differ by race and gender in the GENE sample.

## Methods

### Study design and protocol

**Figure 1:**
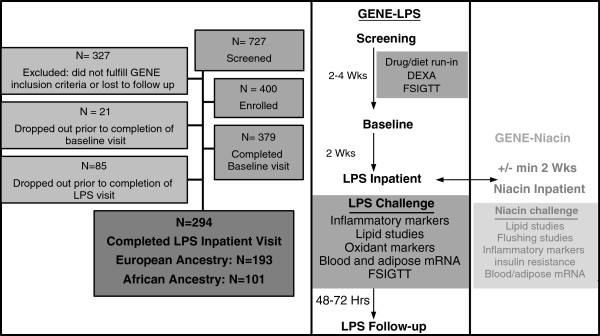
**Design of the genetics of evoked responses to niacin and endotoxemia study.** Healthy volunteers of European and African ancestry participated in the Genetics of Evoked Responses to Niacin and Endotoxemia (GENE) Study. Results are presented here for the 294 individuals that participated in the GENE-LPS component of the study.

The GENE Study recruited healthy volunteers to a University of Pennsylvania (U.Penn) inpatient Clinical and Translational Research Center (CTRC) protocol. Healthy men and non-pregnant non-lactating women, age 18–45, with BMI 18–30 kg/m^2^, and of self-reported African ancestry (AA) or European ancestry (EA) were included. Ancestry was inferred genetically using multidimensional scaling of Affymetrix 6.0 SNP genotypes, and we found high concordance with self-reported ancestry. Of the 294 individuals in GENE-LPS, there were 17 individuals (16 mixed EA/AA, 1 mixed AA/Asian) who did not cluster tightly with either EA or AA, and thus appeared to have mixed ancestry. 12 self-reported EA clustered closer to AA, while 4 self-reported AA clustered with EA, and 1 self-reported AA clustered with neither and appeared to have Asian ancestry. Excluding those 17 individuals did not change the significance of our findings. Exclusion criteria included any current or chronic medical conditions, medication or supplement use, current smoking, and any significant clinical or laboratory abnormalities at screening (Additional file 1). The study design is depicted in Figure 1. Eligible participants (N=294; 52% Female, 35% AA and 65% EA), of 727 undergoing screening, participated in five separate visits, including three outpatient visits and two inpatient visits that addressed separate hypotheses; (i) an endotoxin challenge visit (1 ng/kg *E coli*-derived LPS; U.S. standard reference, lot No. CCRE-LOT-1+2, Clinical Center, Pharmacy Department at the National Institutes of Health, Bethesda MD) (GENE-LPS) and an (ii) a niacin challenge visit (GENE-niacin). Because of complex scheduling, visits did not account for timing relative to the menstrual cycle in women. Findings from GENE-LPS, but not GENE-niacin, are the focus of the current report. The GENE study was approved by U.Penn’s Institutional Review Board (IRB), with regulatory oversight by the FDA (LPS: IND# 5984) and an NIH-appointed data-safety and monitoring board. All subjects provided written informed consent.

#### Baseline visit

Fasting blood samples and anthropometric measurements were taken at a baseline visit that also included DEXA-enhanced whole body analysis (v5.64, Hologic QDR-1000/W; Waltham, MA) for the estimation of body fat mass. All participants received dietary counseling to ensure adherence to a diet representative of the American Heart Association recommendations for healthy living (carbohydrates, 55–60%; protein, 10–15%; and fat, <30%, saturated fat intake 7–10% of total caloric intake/day, total cholesterol <300 mg/day) and subsequently completed dietary records on 3 consecutive days prior to the inpatient visit.

#### LPS inpatient visit

The inpatient LPS visit lasted approximately 40 hours, with a 10-hour overnight acclimatization phase and a 30-hour post-LPS phase, as described [12]. Briefly, following overnight acclimatization, endotoxin (LPS 1 ng/kg) was administered as an intravenous bolus 6:00 am on Day 1. Multiple clinical variables were assessed regularly during the visit, including temperature and blood pressure (recorded and monitored every 15 minutes for the first 8 hours post LPS and hourly thereafter), and heart rate (measured every hour for the first 8 hours post LPS, followed by measurements at 12, 16, and 24 hours post LPS). Complete blood counts (CBC) and a comprehensive metabolic panel were obtained immediately prior to LPS and at 12 and 24-hours post-LPS. Serial blood draws were taken (−15 min, −5 min, and 1, 2, 4, 6, 12, 18, and 24 hours post LPS), for serum and plasma, stored at −80°C, and subsequent measurement of blood biomarkers.

### Laboratory analyses

Plasma levels of tumor necrosis factor alpha (TNFα), interleukin-6 (IL-6), and interleukin-1 receptor agonist (IL-1RA) were measured by high-sensitivity ELISA (Quantikine, R&D Systems; Minneapolis, MN) according to manufacturer’s instructions. The intra- and inter-assay coefficients of variation were: TNF-α, 8.4% and 9.7%; IL-6, 6.4% and 10.5%; IL-1RA, 2.1% and 6.3%; respectively. The lower limits of detection were TNF-α 0.4 pg/mL; IL-6 0.154 pg/mL; IL-1RA 25.4 pg/mL. Plasma lipoproteins and apoproteins were measured enzymatically on a Hitachi 912 Analyzer (Roche Diagnostics; Indianapolis, IN). Serum amyloid A (SAA) and high-sensitivity C-reactive protein (hsCRP) were measured by latex particle-enhanced immunonephelometry on a Behring Nephelometer II Analyzer (Siemens Diagnostics; Munich, Germany).

### Statistical analysis

Our primary analysis examined differences in LPS-induced plasma inflammatory cytokine responses (TNFα, IL-6, IL-1RA) by race and gender. Secondary analyses examined race and gender differences in LPS-induced changes in additional inflammatory and clinical variables as well as race and gender influence on baseline factors prior to LPS. All P values presented are two-sided. In recognition of the analysis of the dual race and gender effects on main outcomes, we have focused only on analyses that demonstrated P<0.025. We did not adjust further because of correlations between variables and because our secondary analyses were performed to complement and inform the primary outcomes. Dependent variables with non-normal distributions were Log-transformed prior to analysis. There were some missing data (<0.3%) across assays due to missed blood draws, or insufficient sample to run all assays (Additional file 1). The analytical models used were robust to missing data. For pre-LPS baseline data analysis of race and gender differences, we compared groups using univariate linear regression models adjusted for race, gender, age and waist to hip ratio (WHR). We initially included a race by gender interaction term to look for interaction effects in the entire sample. Since all interaction P values were >0.1, subsequent models were run to examine the separate effects of race and gender on baseline markers. This was also the case for the LPS-induced responses.

Differences between race and gender groups in LPS-induced responses were similarly analyzed using univariate linear regression models. The primary response variable was the area under the curve (AUC) calculated using the trapezoidal rule, with peak LPS-induced response used in complementary analyses. Incremental models were used to adjust for potential confounders: Model 1 included race, gender, age and WHR along with a race by gender interaction term, Model 2 included race, gender, age, WHR, and the time of LPS visit relative to study initiation, expressed in months; and Model 3 additionally included lipid measurements (HDL, TG) as these are known to differ by race or gender and to possibly relate to inflammatory responses. Correlations between baseline and LPS-induced variables were assessed using Spearman’s rho correlation coefficient (r_s_). Analysis was carried out using SPSS Statistics 19 (IBM, Armonk, NY).

## Results

### Baseline characteristics

The baseline pre-LPS characteristics of the participants who completed the GENE-LPS study are presented in Table 1; Data are stratified by gender and race for ease in interpretation and comparison with published literature. There were small but significant differences in age across groups, with AA being slightly older than EA (P<0.001) and males being older than females (P<0.01). The observed differences by race and gender for most other variables were largely consistent with epidemiological literature providing generalizability for our sample. Blood pressure was higher in AA (P<0.0001), and higher in males (P<0.0001), while heart rate was slightly higher in AA (P<0.05), and lower in males (P<0.0001). Temperature differed by gender, with higher values in females as expected with menstrual physiology [23]. Total fat (e.g. DEXA whole body fat %) and fat distribution (e.g., WHR) differed by gender (P<0.0001) and race (P<0.05) despite similar BMI. HDL-C and apoA-I were higher in women (P<0.001) while HDL-C levels were higher (P<0.05) and TG levels lower (P<0.0001) in AA than in EA.

**Table 1:** Baseline characteristics of participants in the Genetics of Niacin and Endotoxemia (GENE-LPS) study

	**Male EA N=102**	**Male AA N=41**	**Female EA N=91**	**Female AA N=60**	**Race**	**Gender**
	**Median (IQR)**	**Median (IQR)**	**Median (IQR)**	**Median (IQR)**	P value*	P value*
**Age (years)**	24 (6)	28 (16)	24 (6)	23.5 (10)	0.0004	0.0016
**BMI (kg/m**^**2**^**)**	23.65 (3.9)	25.2 (4.9)	23 (3.7)	22.4 (5.6)	0.24	0.07
**Body fat % (Trunk)**	14.7 (8)	15.4 (9.2)	24 (9.6)	20 (12.9)	0.08	<0.0001
**Body fat % (whole body)**	16 (7)	17.2 (8)	28.7 (7.3)	24.7 (10)	0.03	<0.0001
**Waist circumference (cm)**	84.5 (10.6)	87 (12.5)	83 (13.5)	78.5 (12.8)	0.07	0.0059
**Hip Circumference (cm)**	97 (8)	98 (10.5)	98 (12)	97 (8)	0.65	0.67
**Waist:Hip Ratio**	0.87 (0.1)	0.86 (0.1)	0.85 (0.1)	0.81 (0.1)	0.0006	<0.0001
**Weight (kg)**	74.9 (14.3)	77.6 (17.5)	62.5 (14.2)	64.1 (12.7)	0.45	<0.0001
**Systolic Blood Pressure (mmHg)**	112 (15.5)	118 (12)	108 (11)	112.5 (19)	<0.0001	0.0001
**Diastolic Blood Pressure (mmHg)**	64 (10)	73 (9.5)	61 (12)	66 (13)	<0.0001	0.0006
**Heart rate (bpm)**	60 (11)	61 (14.5)	65 (13)	69.5 (10.8)	0.03	<0.0001
**Temperature (°C)**	36.5 (0.3)	36.5 (0.4)	36.6 (0.4)	36.7 (0.4)	0.22	<0.0001
**Total Cholesterol (mg/dl)**	146 (44.5)	161 (46.5)	152 (38)	150.5 (39.5)	0.52	0.81
**HDL-C (mg/dl)**	47 (14)	49 (28)	52 (16)	58 (17)	0.02	0.001
**LDL-C (mg/dl)**	81 (34)	93.8 (33.1)	76 (35.2)	78.9 (35.05)	0.48	0.06
**Triglyceride (mg/dl)**	71.5 (37)	61 (36.5)	69 (38)	51 (23.5)	<0.0001	0.35
**Apo A1 (mg/dl)**	119.5 (25)	120 (43)	131 (33)	130 (36.5)	0.60	0.0002
**Apo B (mg/dl)**	60.5 (22)	69 (24)	60 (24)	59.5 (19)	0.88	0.22
**WBC (x10-3/ul)**	5.9 (2.1)	5.1 (1.2)	6.4 (1.8)	5.6 (2.8)	0.03	0.001

*BP*, Blood Pressure; *BMI*, Body Mass Index; *Apo A1*, Apolipoprotein A1; *Apo B*, Apolipoprotein B; *HDL-C*, high-density lipoprotein cholesterol; *LDL-C*, low-density lipoprotein cholesterol; *WBC*, white blood cell count.

*P values are from linear regression models run on log-transformed variables for non-normally distributed variables. Models adjusted for Age and Waist:Hip ratio (WHR) (No WHR adjustment in adiposity comparisons).

Consistent with published literature [24,25], baseline inflammatory markers differed by gender, with higher circulating levels of WBC (P=0.001) (Table 1), IL-6 (P=0.005), IL-1RA (P<0.0001) in women than men (Table 2). Although levels in EA and AA also were consistent with published literature [7,25], differences were statistically significant only for lower WBC (P<0.05) (Table 1) and higher plasma IL-6 (P=0.001) in AA vs. EA (Table 2).

**Table 2:** Baseline and evoked inflammatory biomarker responses to endotoxemia

	**Male EA N=102**	**Male AA N=41**	**Female EA N=91**	**Female AA N=60**	**Race**	**Gender**
	**Median (IQR)**	**Median (IQR)**	**Median (IQR)**	**Median (IQR)**	P value*	P value*
**TNFα (pg/mL) Baseline**	1.08 (0.6)	1.03 (0.6)	1.06 (0.6)	1.13 (0.9)	0.64	0.66
**Peak**	43.5 (44)	37.3 (34)	43.2 (66)	34.0 (39)	0.42	0.55
**AUC**	156.7 (152)	123.4 (117)	151.4 (215)	116.4 (124)	0.18	0.12
**IL-6 (pg/mL) Baseline**	1.9 (2.1)	2.3 (2)	2.3 (1.7)	2.9 (2.2)	0.0013	0.0047
**Peak**	138.3 (158)	127.0 (134)	162.6 (195)	143.7 (149)	0.89	0.08
**AUC**	403 (403)	299 (362)	414 (410)	364 (300)	0.51	0.36
**IL-1RA (pg/ml) Baseline**	106.9 (53)	91.4 (55)	121.0 (64)	121.6 (67)	0.64	<0.0001
**Peak**	42843 (41293)	27676 (43350)	44073 (50058)	29789 (35435)	0.002	0.89
**AUC**	133260 (127732)	88663 (131287)	136600 (144853)	87536 (97476)	0.0007	0.96
**CRP (mg/L) Baseline**	0.34 (0.6)	0.54 (0.9)	0.62 (1.4)	0.71 (1.7)	0.40	0.11
**Peak**	17.8 (13)	14.3 (11)	15.9 (12)	10.8 (8.3)	0.005	0.12
**AUC**	196 (111)	164 (107)	171 (116)	127 (84)	0.002	0.02
**SAA (mg/L) Baseline**	2.8 (0.2)	2.8 (0.6)	2.8 (2.3)	2.8 (1.6)	0.48	0.25
**Peak**	91.0 (53)	74.4 (62)	80.7 (51)	57.7 (58)	0.16	0.02
**AUC**	1073 (602)	858 (710)	939 (582)	773 (572)	0.03	0.04

*TNFα*, Tumor necrosis Factor alpha; *IL-6*, Interleukin 6; *IL-1RA*, Interleukin 1 receptor agonist; *CRP*, C-reactive protein; *SAA*, Serum Amyloid A; *AUC*, area under the curve, calculated using the trapezoidal rule. *P values are from linear regression models run on log-transformed variables for non-normally distributed variables. Models adjusted for Age and Waist:Hip ratio (WHR).

### Clinical responses to endotoxemia have similar patterns across gender and race

As expected [12,15,16], LPS (at 1ng/kg IV) was well tolerated by subjects, and induced a clinical response characterized by changes in temperature, heart rate and blood pressure (all P<0.0001; e.g., mean peak post-LPS increase of 0.9°C, 27bpm and 20/22 mmHg for temperature, heart rate and diastolic/systolic blood pressure respectively), (Additional file 1: Figure S1 A-D). Temperature and heart rate dipped at 6 hours post-LPS coincident with a per-protocol lunch at midday. No significant race-by-gender interaction was observed for these LPS-induced clinical responses (all P values >0.1 for AUC and peak). Further, there were no significant differences by race or gender in the response of these clinical variables to LPS in models adjusted for age and WHR or further adjusted for their pre-LPS values.

### Inflammatory biomarker responses to endotoxemia are lower in individuals of African ancestry

**Figure 2:**
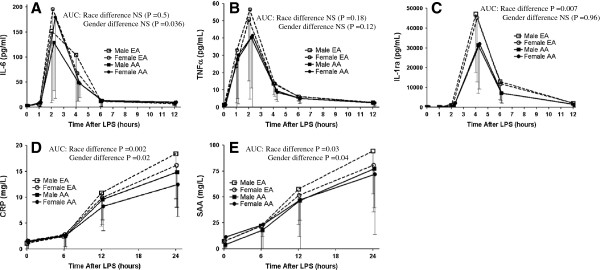
**Race and gender influence on circulating inflammatory biomarker responses to endotoxemia.** European ancestry participants have a higher peak inflammatory response than African ancestry individuals for (**A**) IL-6, (**B**) TNFα, (**C**) IL-1RA, (**D**) CRP, and (**E**) SAA.

On average, there was a robust acute inflammatory response to LPS with increases in circulating cytokines TNFα (~50-fold, average peak at 2 hours), IL-6 (~75-fold, average peak at 2 hours) and IL-1RA (~350-fold, average peak at 4 hours) as well as the acute phase proteins, CRP (~30 fold, average peak 24 hours) and SAA (~25 fold, average peak 24 hours) (P<0.0001 for all compared to baseline values) (Figure 2A-E).

Despite a trend toward higher pre-LPS levels of several cytokines in AA individuals, LPS-induction of cytokines was significantly lower in AA than EA individuals (Table 2) in models adjusted for age, gender, WHR and timing of LPS visit in months relative to study initiation. For example, the mean of individual fold increases in IL1RA levels (pg/ml) from baseline to peak were 443 vs. 351-fold in EA and AA males and 386 vs. 268-fold in AA vs. EA females (P=0.002) (Figure 2C). Both TNFα and IL-6 (Figure 2A and 2B) followed the same pattern, with lower induction in AA, although this did not reach statistical significance. Similarly, compared to EA, AA individuals had a significantly lower LPS-induced CRP (P=0.002) and SAA (P=0.03) responses. Additional adjustment for baseline levels of TG and HDL-C, surrogates for lipoproteins that can modulate LPS bioavailability and activity [26,27] that also differ by race, did not attenuate race-differences in cytokine and acute phase responses (not shown).

There was a modest gender difference in CRP response, with higher peak and AUC in males (P=0.02); the mean of individual fold increases from baseline to peak in CRP levels (mg/L) were 51 vs. 28-fold in EA and AA males and 31 vs. 25-fold in EA vs. AA females (Figure 2D). WBC increased slightly, but significantly, in all subjects at 12-hours post-LPS (P=0.0001), with no statistically significant race or gender differences in the response, although sampling frequency may have limited the ability to detect differences (Additional file 1: Figure S2).

### Baseline cytokine levels are poorly correlated with peak inflammatory responses

Baseline levels of inflammatory cytokines were only weakly correlated with each other (r_s_ 0.1-0.3, P<0.01), and did not correlate with peak inflammatory levels (r_s_ <0.1, all P>0.05). In contrast, peak cytokine levels correlate highly with each other (r_s_ 0.76-0.85, all P<0.0001), and also with peak acute phase proteins (r_s_ 0.3-0.6, all P<0.0001) (Additional file 1: Table S1), indicating greater convergence and consistency of evoked responses compared with baseline measurements. These patterns of correlation were consistent across race.

## Discussion

Innate immunity plays a key role in the acute defense against pathogens and has emerged as an important modulator of chronic cardiometabolic diseases including T2DM and atherosclerosis. Here, as part of the GENE study, we utilized experimental endotoxemia in healthy individuals to probe demographic influences on the extent and nature of the induced acute inflammatory response. Compared to EA, we found a pattern of lower inflammatory biomarker responses in AA participants. Although temperature and hemodynamic responses did not differ by race, these parameters are subject to homeostatic regulation that may blunt differential race influences of inflammation on these key regulators of host survival. In contrast, the liver production of acute-phase proteins, CRP and SAA, which are not subject to the same homeostatic regulation, demonstrated substantial difference by race, tracking the pattern observed for the acute cytokine response. The functional role of these cytokines in immune responses, as well as the established utility of CRP and SAA as biomarkers of cardiometabolic disease risk [9,10], suggest clinically relevant consequences of these observed race-differences in response to endotoxemia.

Our data provide several novel insights beyond epidemiological observations. First, in the GENE study, the LPS-evoked inflammatory cytokine response was higher in EA compared with AA. In support of our finding, one small study of Duffy antigen effect on endotoxin response found that Duffy-negative males of African descent had lower inflammatory response (plasma monocyte chemoattractant protein-1) than Duffy-positive males of European descent [28], although separate effects of race and Duffy antigen were not addressed. Second, we identified an interesting paradox in inflammatory biomarker response compared to resting biomarker levels. In epidemiological studies, plasma levels of cytokines and inflammatory markers that predict cardiometabolic disease [10,29] differ by race with higher resting levels of IL-6 and CRP in AA compared to EA [7,30,31]. Although we observed similar trends for higher pre-LPS levels of several biomarkers in AA in the GENE study, there was an opposite response to endotoxemia, with lower peak levels in AA compared to EA. For example, in AA relative to EA, the LPS-induced response of plasma IL-1RA and CRP were lower, whereas baseline levels tended to be higher. Third, regardless of race, the LPS-induced cytokine responses had greater correlations with each other and with the subsequent increases in acute-phase proteins than the correlations observed for the pre-LPS cytokines with baseline biomarkers or with LPS-induced responses. This suggests that the evoked inflammatory response may reflect more accurately the biochemical and clinical consequences of acute activation of innate immunity than resting levels of inflammatory markers. Thus, measuring resting levels of inflammatory biomarkers in epidemiological studies may provide limited insight into the stress responses that are most relevant to disease related activation of innate immunity.

We interpret these findings cautiously because we do not yet know whether inflammatory responsiveness to endotoxemia has clinical relevance in disease risk prediction. Further, it is plausible that “baseline” levels of inflammatory biomarkers may reflect an ongoing low-grade inflammation that is relevant to the development of cardiometabolic diseases while the acute responses to LPS during experimental endotoxemia may be more relevant to acute inflammatory disorders like sepsis, SIRS, or other infectious diseases. However, genetic variation in TLR4 that modulates inflammatory response and sepsis has been associated with reduced risk of atherosclerosis and CVD [22] supporting the relevance of modeling acute TLR4 signaling responses to risk of chronic cardiometabolic disease.

The mechanisms of race-differences in inflammatory responses are uncertain but likely complex. One possibility is that the pattern observed in AA (higher pre-LPS but reduced induced cytokines) reflects chronic conditioning or priming of innate immunity in AA individuals resulting in an attenuated responsiveness during inflammation [32]. Lower peak levels of the inflammatory-modulating IL-1RA in AA suggest that there may be a blunted resolution response as well as attenuated pro-inflammatory signaling. A teleological basis for the race differences may lie in distinct evolutionary pathogenic pressures in African populations compared with those that migrated to northern geographic regions [33,34] leading to race and geographic differences in the inflammatory response to innate immune antigens including those modulated by diet and lifestyle factors [28,35]. These concepts, however, require further testing in experimental, genomic and clinical studies.

We observed modest gender differences in the response to LPS with a greater CRP and SAA response in males compared to females. However, not all responses were significant or consistent e.g., peak IL-6 showed a non-significant trend toward higher response in females. Previous small studies also have shown mixed results; CRP and TNFα were increased in response to LPS in females compared with males in a European study (N=30) [36] but no differences in cytokines were seen in a US study (N=24) despite differences in temperature response [37]. The GENE study recruited a much larger sample (N=294) and included race-stratified analyses. Overall, our findings suggest small, although nominally statistically significant, differences by gender in some inflammatory responses. The gender influence was less than those observed for race and is of uncertain clinical significance given modest differences and inconsistent findings across biomarkers.

Although our data did reveal consistent racial differences in inflammatory responses, the observed race-differences were modest (< 2-fold for most biomarkers) relative to the inter-individual differences in response in both EA and AA (e.g., 100-fold differences in some plasma biomarker responses between the 95^th^ and 5^th^ percentile responders). Such large inter-individual differences suggest that a relatively small portion of the LPS responses is attributable to race *per se*. Application of unbiased genetic approaches in the GENE study sample may provide novel insights into the genetic basis of innate immune responses in human and also might help identify genomic influences specific to EA and AA populations.

Our study has several unique strengths but also limitations. To our knowledge, this is the largest human experimental endotoxemia protocol published to date and the only one specifically designed to probe differences between AA and EA in the inflammatory and metabolic responses to activation of innate immunity. A distinct advantage of the model is that it controls the temporal and directional activation of innate immunity and downstream responses and thus eliminates concerns regarding reverse causation and confounding when studying patients with established chronic disease. Experimental endotoxemia is a model of activation of innate immunity that partly approximates the acute pathophysiology of sepsis syndromes. Several lines of evidence also suggest that this model may be informative in cardiometabolic disease [11,14] although we acknowledge that it is unknown whether inflammatory responsiveness to endotoxemia is relevant to clinical disease prediction. Observational data show that sepsis and chronic infection [38,39] induce insulin resistance, glucose intolerance and lipid derangement resembling that observed in obesity, T2DM and atherosclerosis. We and others have shown that experimental endotoxemia induces insulin resistance [13,14], adipose tissue inflammation [40] and atherogenic lipoprotein changes [21]. TLR4 is directly implicated in diet-induced obesity and atherosclerosis [19] through studies in mouse models, while dietary modulation of the gastrointestinal biome has been shown to influence blood levels of LPS [41]. Atherosclerotic plaque contains microbes, likely oral and gut-derived [42], while blood microbial load may be predictive of development of diabetes [43] providing additional support for a link between the immune response to bacteria and cardiometabolic disease.

## Conclusions

We describe the GENE study and report the novel finding of differences in LPS-induced inflammatory responses in individuals of African vs. European ancestry. A lower inflammatory response in AA participants during endotoxemia may contribute to and be predictive of the observed race-differences in clinical disorders characterized by activation of innate immunity. Genomic and functional studies are required to define the underlying mechanisms of these race-differences and to provide prognostic or therapeutic hypotheses for testing in clinical inflammatory disorders.

## Abbreviations

GENE: Genetics of evoked responses to niacin and endotoxemia; EA: European ancestry; AA: African ancestry; LPS: Lipopolysaccharide; CHD: Coronary heart disease.

## Competing interests

The authors declared that they have no competing interest.

## Authors’ contributions

JFF and MPR drafted the manuscript, and all authors reviewed and approved the final version of the manuscript. Subject recruitment and data collection was carried out by JFF, PNP, RS, CKM, RG, PSN, HU, NNM, RS, SRM. Data were analyzed and interpreted by JFF, KJP, MPR. MPR was responsible for study conception and design. All authors read and approved the final manuscript.

## Supplementary Material

Additional file 1 Supplementary methods, tables and figures. Click here for file
