# Supplementary material for: Race and gender variation in response to evoked inflammation
Source: J Transl Med. 2013 Mar 12;11:63. doi: 10.1186/1479-5876-11-63 (PMC3636014; doi:10.1186/1479-5876-11-63)
Supplement: Additional file 1 — Supplementary methods, tables and figures. [file 1479-5876-11-63-S1.docx]

**Supplementary Material**

**Race and gender variation in response to evoked inflammation**

Jane F Ferguson et. al.

**Supplementary Methods**

**Missing data**

For TNFα there were 3 missing data points across 3 individuals (0.2%). For IL-6 there were 4 missing data points across 4 individuals (0.2%). For IL1RA, there were 5 missing data points, across 4 individuals (0.3%). For SAA there were 3 missing data points across 3 individuals (0.25%). For CRP there was one missing data point, in one individual (0.1%). There were 13 subjects missing DEXA body composition analysis.

**GENE Study Exclusion Criteria**

1. Known clinically manifest atherosclerotic cardiovascular disease, including coronary disease, cerebrovascular disease, or peripheral vascular disease

2. History of diabetes mellitus

3. Fasting glucose >126mg/dL at screening

4. History of a non-skin malignancy within the previous 5 years

5. Renal insufficiency as defined by creatinine > 1.5 mg/dl at Screening Visit

6. History of liver disease or abnormal LFTs (AST, ALT, Alk. Phos, GGT > 1.5x ULN; bilirubin > 2x ULN) at Screening Visit

7. Men who are unwilling to limit alcohol consumption to < 14 alcoholic drinks per week or < 4 alcoholic drinks per occasion (AMA / NIAAA criteria for “at risk” usage levels) while participating in the study

8. Women who are unwilling to limit alcohol consumption to < 7 alcoholic drinks per week or < 3 alcoholic drinks per occasion (AMA / NIAAA criteria for “at risk” usage levels) while participating in the study

9. Total white blood cell count less than or equal to 3.0 THO/µL

10. Hemoglobin below 11.0 g/dL

11. Any major active rheumatologic, pulmonary, or dermatologic disease or inflammatory condition or minor active infection

12. History of HIV positive

13. First degree family history of premature cardiovascular disease event (father or brother if diagnosed at before 55 years of age; mother or sister if diagnosed before 65 years of age)

14. Patients who have undergone any organ transplant

15. Individuals who currently use tobacco products or have done so in the previous 30 days

16. Treatment with aspirin, NSAIDs, COX-2 inhibitors, steroids or any immunomodulatory therapy 2 weeks prior to the Screening Visit

17. Treatment with statins, fibrates or niacin 4 weeks prior to the Screening Visit.

18. Current daily use of Vitamin C > 1000 mg, Beta carotene > 1000 IU, vitamin A > 5000 IU, vitamin E > 400 IU, and selenium > 200 mcg

19. Positive urine pregnancy at the Screening Visit

20. Participation in another clinical trial within the previous 6 weeks prior to the Screening Visit

21. Poorly controlled blood pressure (BP > 160/110) or on any anti-hypertensive medications.

22. A diagnosis of metabolic syndrome using updated 2004 NCEP ATPIII criteria.

23. A history of severe lactose intolerance (e.g., intolerance of any milk intake)

24. Any medical condition or abnormal laboratory value that is judged clinically significant by an investigator

**Figure S1. Clinical responses to endotoxemia by race and gender.** (A) Temperature, (B) heart rate, (C) Systolic Blood Pressure and (D) Diastolic Blood Pressure

**A**


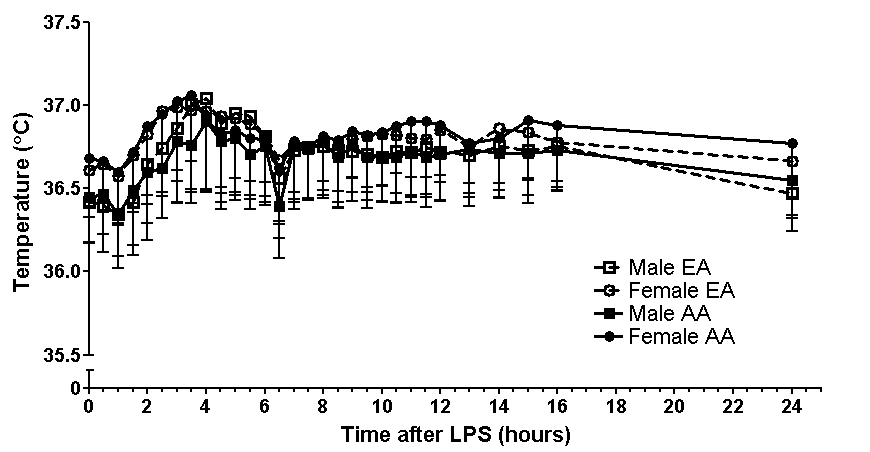


**
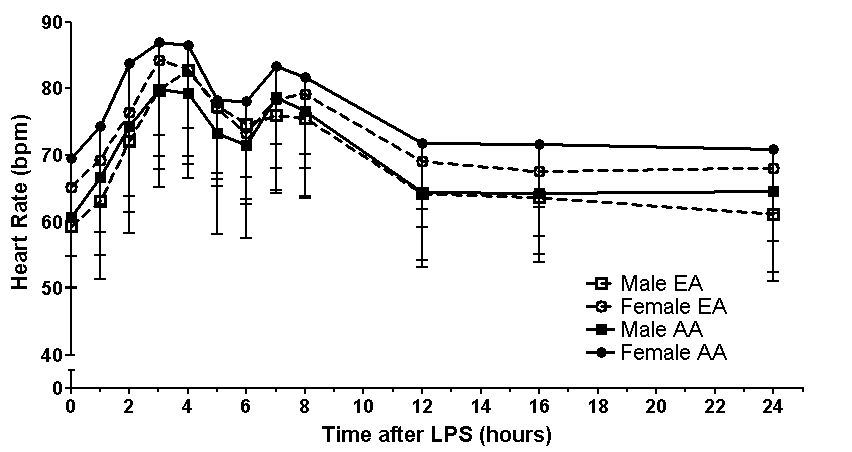
**

**B**

**
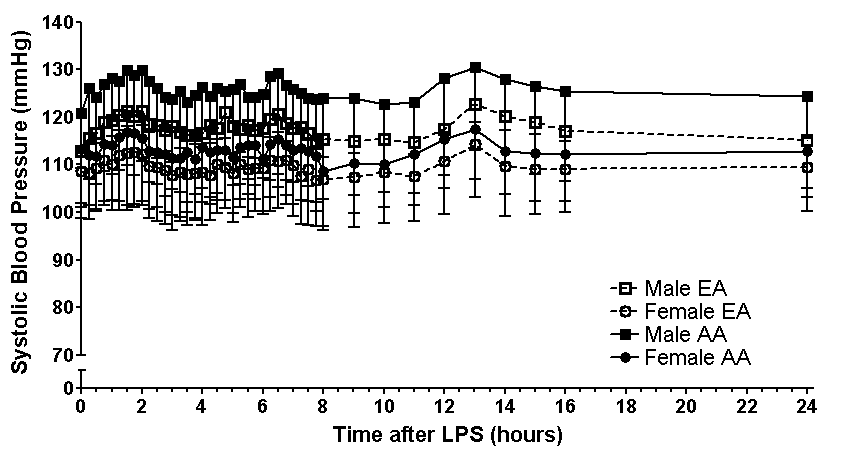
**

**C**

**
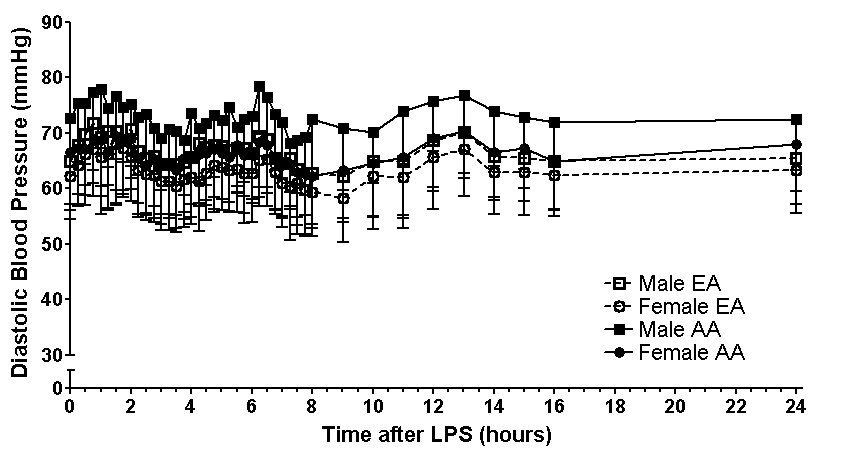
**

**D**

**Figure S2. WBC response to endotoxemia by race and gender**

**
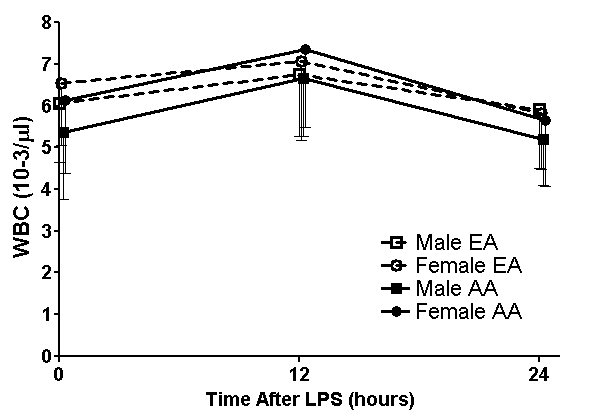
**

**Table S1. Correlation between baseline and peak inflammatory markers in (A) African ancestry and (B) European ancestry.** Relative to baseline values, peak levels of inflammatory cytokines had greater correlations with each other and with peak CRP and SAA than the correlation observed for baseline levels of cytokines with each other and with LPS-induced responses. Correlations were assessed using Spearman’s rho correlation coefficient.

| **African Ancestry** | | **Baseline** | | | | | | **Peak** | | | | | | |
| --- | --- | --- | --- | --- | --- | --- | --- | --- | --- | --- | --- | --- | --- | --- |
|  |  |  | **IL-1RA pg/ml** | **IL-6 pg/ml** | **TNFα pg/ml** | **CRP mg/L** | **SAA mg/L** | **IL-1RA pg/ml** | **IL-6 pg/ml** | **TNFα pg/ml** | **CRP mg/L** | **SAA mg/L** | **Temp**  **˚F** | **HR**  **bpm** |
| **Baseline** | **IL-1RA pg/ml** | rho | 1.00 | .23^*^ | .21^*^ | .34^**^ | .26^*^ | .038 | -.031 | .010 | .13 | -.075 | .039 | .059 |
|  |  | P | . | .020 | .033 | .001 | .010 | .71 | .76 | .92 | .19 | .46 | .69 | .560 |
|  | **IL-6 pg/ml** | rho | .23^*^ | 1.00 | .050 | .14 | .24^*^ | .091 | .22^*^ | .046 | .12 | -.028 | .22^*^ | .27^**^ |
|  |  | P | .020 | . | .62 | .16 | .014 | .36 | .029 | .65 | .23 | .78 | .026 | .007 |
|  | **TNFα pg/ml** | rho | .21^*^ | .050 | 1.00 | .11 | .041 | -.11 | .006 | -.043 | .043 | -.21^*^ | .008 | -.104 |
|  |  | P | .033 | .62 | . | .27 | .68 | .29 | .96 | .67 | .67 | .037 | .93 | .30 |
| **Peak** | **IL-1RA pg/ml** | rho |  |  |  |  |  | 1.00 | .79^**^ | .84^**^ | .61^**^ | .44^**^ | .26^**^ | .19 |
|  |  | P |  |  |  |  |  | . | .000 | .000 | .000 | .000 | .010 | .055 |
|  | **IL-6 pg/ml** | rho |  |  |  |  |  | .79^**^ | 1.00 | .76^**^ | .49^**^ | .36^**^ | .35^**^ | .33^**^ |
|  |  | P |  |  |  |  |  | .000 | . | .000 | .000 | .000 | .000 | .001 |
|  | **TNFα pg/ml** | rho |  |  |  |  |  | .84^**^ | .76^**^ | 1.00 | .56^**^ | .34^**^ | .22^*^ | .23^*^ |
|  |  | P |  |  |  |  |  | .000 | .000 | . | .000 | .000 | .031 | .021 |

**A.**

* Correlation is significant at the 0.05 level (2-tailed); ** Correlation is significant at the 0.01 level (2-tailed)

IL-1RA, Interleukin 1 receptor agonist; IL-6, Interleukin 6; TNFα, Tumor necrosis Factor alpha; CRP, C-reactive protein; SAA, Serum Amyloid A; Temp, Temperature; HR, heart rate

**B.**

| **European Ancestry** | | **Baseline** | | | | | | **Peak** | | | | | | | |
| --- | --- | --- | --- | --- | --- | --- | --- | --- | --- | --- | --- | --- | --- | --- | --- |
|  |  |  | **IL-1RA pg/ml** | **IL-6 pg/ml** | **TNFα pg/ml** | **CRP mg/L** | **SAA mg/L** | | **IL-1RA pg/ml** | **IL-6 pg/ml** | **TNFα pg/ml** | **CRP mg/L** | **SAA mg/L** | **Temp**  **˚F** | **HR**  **bpm** |
| **Baseline** | **IL-1RA pg/ml** | rho | 1.00 | .23^**^ | .18^*^ | .30^**^ | .24^**^ | | .053 | .100 | .031 | .090 | .028 | .059 | .091 |
|  |  | P | . | .001 | .011 | .000 | .001 | | .47 | .17 | .67 | .21 | .70 | .42 | .21 |
|  | **IL-6 pg/ml** | rho | .23^**^ | 1.00 | .32^**^ | .30^**^ | .24^**^ | | -.028 | .015 | -.072 | .017 | -.12 | .019 | .03 |
|  |  | P | .001 | . | .000 | .000 | .001 | | .69 | .83 | .32 | .82 | .11 | .79 | .68 |
|  | **TNFα pg/ml** | rho | .18^*^ | .32^**^ | 1.00 | .16^*^ | .13 | | -.082 | -.083 | -.096 | -.12 | -.13 | -.090 | -.049 |
|  |  | P | .011 | .000 | . | .024 | .078 | | .26 | .25 | .18 | .094 | .066 | .22 | .49 |
| **Peak** | **IL-1RA pg/ml** | rho |  |  |  |  |  | | 1.000 | .80^**^ | .86^**^ | .57^**^ | .59^**^ | .34^**^ | .29^**^ |
|  |  | P |  |  |  |  |  | | . | .000 | .000 | .000 | .000 | .000 | .000 |
|  | **IL-6 pg/ml** | rho |  |  |  |  |  | | .804^**^ | 1.00 | .79^**^ | .61^**^ | .57^**^ | .43^**^ | .41^**^ |
|  |  | P |  |  |  |  |  | | .000 | . | .000 | .000 | .000 | .000 | .000 |
|  | **TNFα pg/ml** | rho |  |  |  |  |  | | .855^**^ | .79^**^ | 1.00 | .56^**^ | .47^**^ | .34^**^ | .29^**^ |
|  |  | P |  |  |  |  |  | | .000 | .000 | . | .000 | .000 | .000 | .000 |

* Correlation is significant at the 0.05 level (2-tailed); ** Correlation is significant at the 0.01 level (2-tailed)

IL-1RA, Interleukin 1 receptor agonist; IL-6, Interleukin 6; TNFα, Tumor necrosis Factor alpha; CRP, C-reactive protein; SAA, Serum Amyloid A; Temp, Temperature; HR, heart rate
